# Revolution in Health Care: How Will Data Science Impact Doctor–Patient Relationships?

**DOI:** 10.3389/fpubh.2018.00099

**Published:** 2018-04-03

**Authors:** Ivan Lerner, Raphaël Veil, Dinh-Phong Nguyen, Vinh Phuc Luu, Rodolphe Jantzen

**Affiliations:** ^1^UMR8156 Institut de recherche interdisciplinaire sur les enjeux sociaux Sciences sociales, Politique, Santé (IRIS), Paris, France; ^2^Sorbonne Université, UPMC Univ Paris 06, Paris, France; ^3^Univ Paris Diderot, Sorbonne Paris Cité, Faculté de médecine, Paris, France; ^4^Université Paris Est, Faculté de médecine, Créteil, France

**Keywords:** doctor–patient relationship, data science, artificial intelligence, machine learning, digital health

Over the last decade, a technical revolution has taken place in several industrial sectors, starting with internet companies. The computerization and interconnection of a wide variety of services and devices has facilitated the collection and storage of data, which has since increased by several orders of magnitude. The exploitation of these data has completely reshaped some services, such as Internet advertising, which has become largely personalized, while bringing with it its fair share of privacy issues. As data management and analysis have become central to many businesses, computer scientists have been called upon to provide tools capable of extracting knowledge from ever-growing, structured and unstructured databases. In this context, a paradigm shift occurred in data analysis as more data became available; with deep learning, data-driven approaches are nowadays often surpassing domain-specific approaches (1). Indeed, in very diverse predictive tasks, such as machine translation (2), object recognition (3) or speech recognition (4), general purpose models such as artificial neural networks have outperformed advanced algorithms developed by experts with domain-specific knowledge. Additionally, these machine learning algorithms have often reached experts’ performance level at various tasks, including medical diagnosis (5–8). However, these great successes have often been achieved at great expense: the acquisition of a large amount of structured and unstructured data.

Big health-care data are already a reality; academias, industries, insurance agencies, and public health systems struggle to adapt their infrastructure to a data volume whose size is doubling every 12–14 months (9). Such storage systems are also challenging in terms of accessibility, ownership, and privacy issues (10). Still, medical uses remain mainly in the field of research, aiming to provide information about patients’ conditions by analyzing massive amounts of data and assisting with decision-making. Within hospitals, some data-driven softwares are being developed to identify patients at high risk of hospital mortality (11), while others predict patient affluence and/or waiting times in emergency departments (12). Outside the hospital, data-driven applications are also flourishing in various fields, such as telemonitoring systems, implementing advanced prediction of asthma exacerbations (13), or automatic detection of falls in the elderly population (14). Moreover, precision medicine aims to provide a personalized recommendation of the optimal treatment for each patient, relying on the analysis of large heterogeneous datasets, including imaging, genomics, or various biological values extracted from electronic health records. This framework can be applied in many areas of medicine, such as radiation oncology (15), psychiatry (16), and infectious diseases (17). While these developing medical applications will require rigorous clinical validation, many should find their way into daily clinical practice over the next few decades (Figure 1). How will such innovations impact clinicians and their relationships with patients?

**Figure 1:**
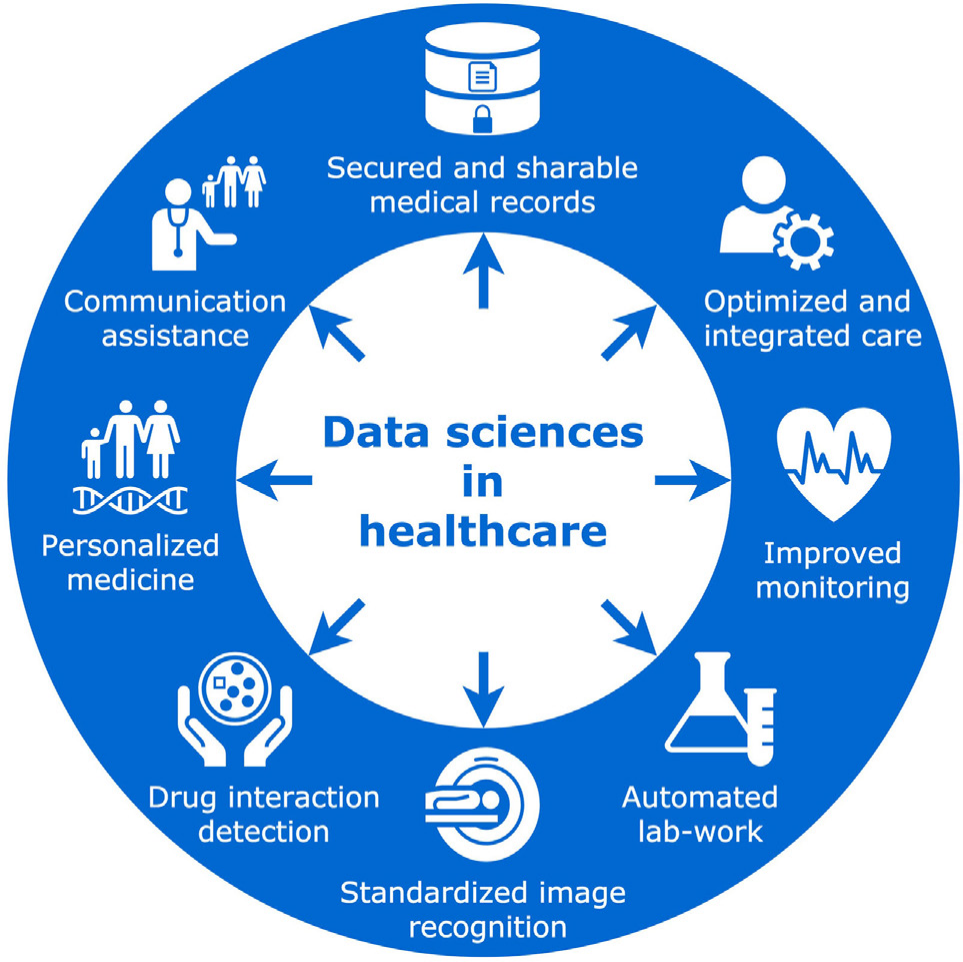
Revolution in healthcare.

First, let us explore what was driving this relationship before data science found its way into health care. Traditionally, the work of physicians is a balancing act between technical expertise and human interpersonal skills. On the one hand, medical doctors aim to improve their knowledge to diagnose diseases more accurately and recommend optimal treatment for each specific health condition. On the other hand, they strive to be more empathetic toward patients, taking into account their psychosocial background and cultural beliefs. Today, most doctors know that engaging in a dialog with each patient is critical in delivering adapted information, improving adherence to treatment, and ensuring an understanding of their condition. However, in the mid-twentieth century, with the biomedical revolution reaching its peak, medicine became increasingly efficient but also more technical. In addition, the trust between doctors and patients has always faced multiple challenges, including language and cultural barriers, potentially threatening alternative medicine, or more recently the decreasing importance of family practice (18). In light of these evolutions, data science is not going to be the first phenomenon to challenge physicians’ ability to adapt and rethink their profession (Figure 2).

**Figure 2:**
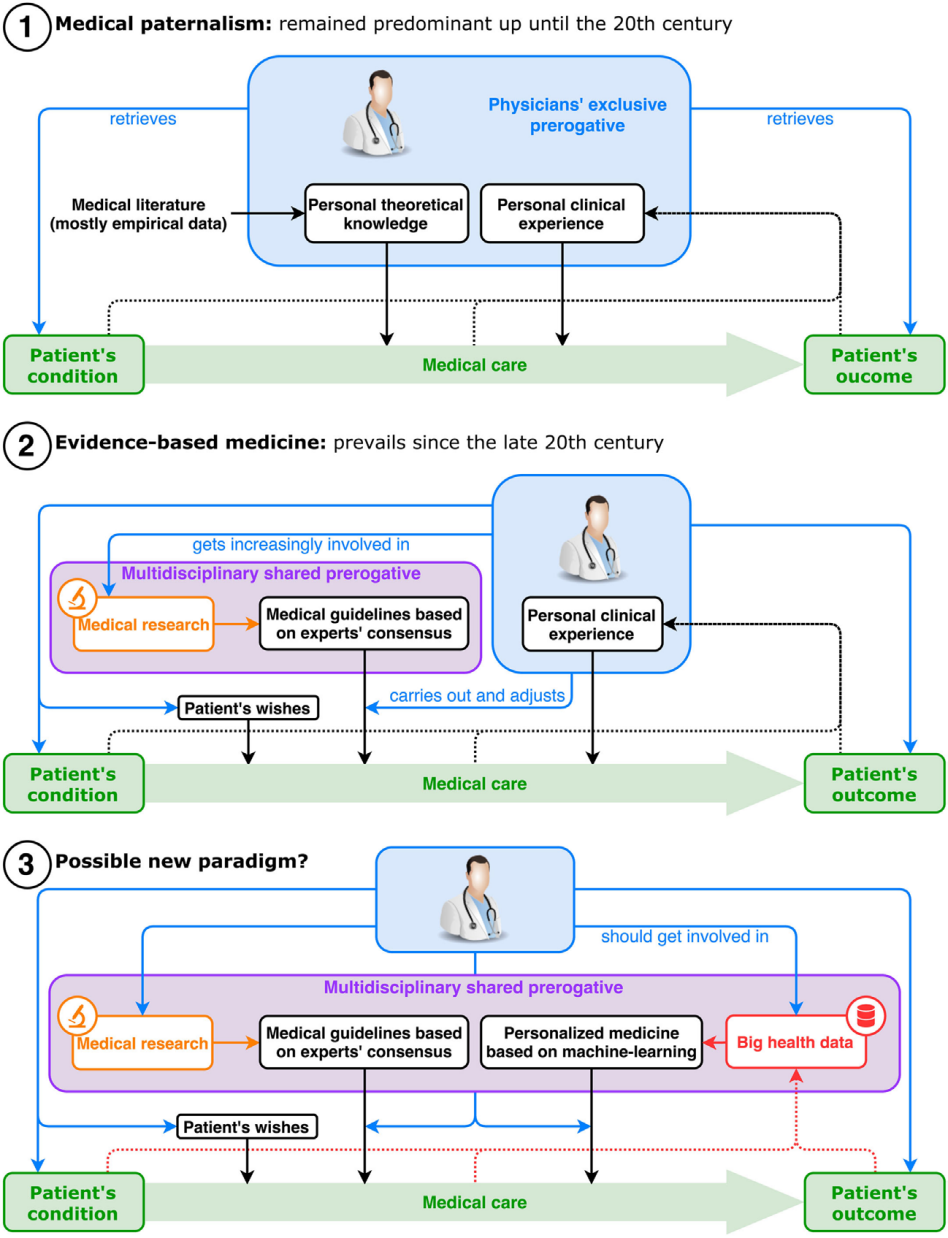
Evolution of the physician’s role in healthcare.

This rapid increase in technical knowledge regarding medicine and biology brought, as a corollary, the development of methodically conceived standardized guidelines for physicians to apply. In an effort to push evidence-based medicine onto the field, health authorities worked with experts to offer, promote, and soon enforce the strict application of these guidelines by more and more professionals (19). This phenomenon aimed to increase health-care quality, equity, and security to patients, is sometimes criticized for being too rigid to apply individually and for reducing the autonomy of physicians (20). In France for instance, it is not yet mandatory to follow such guidelines, but physicians may be held responsible for not being able to justify their deviation from them. Some physicians have now traded this decision-making privilege with much more implication in research, public health, the elaboration process of these guidelines, or simply the exploration of new areas of expertise in their field. And all these new tasks, including the ones that came along with the standardization of health care, have taken up an increasing amount of personal and collective efforts, leaving ever less time to dedicate to the improvement of doctor–patient relationships.

Alongside these changes, a cultural movement inspired by the social sciences and humanities appeared in North America in the late 1970s, focusing on the importance of human skills in clinical practice (21). One trigger may have been the realization that patient adherence to treatment was much lower under real conditions than in clinical trials, sometimes making efforts to improve treatment efficacy irrelevant (22, 23). As end-of-life situations became more and more medicalized, patients’ expectations of care attitudes often contrasted with physicians’ healing behaviors. This led to the development of palliative care (24, 25), with a special focus toward patients’ reported experience. Patient empowerment through stronger patient organizations has also led to more patient-centered care (26, 27). Additionally, attention to patient choice, consent, psychosocial context, and cultural beliefs has become increasingly important in research (28, 29) and clinical practice, so much so that it became a key element in the concept of Evidence-Based Medicine. Medical schools then started training their students in medical ethics, patient communications, therapeutic education, and narrative medicine (30, 31). In most developed countries, laws have been amended to recognize patients’ right to refuse treatment or to be informed of their medical conditions, thus striking a blow to the long-lasting paradigm of medical paternalism. How will these cultural movements respond to the arrival of medical data-driven applications, a new set of technologies aimed at further “dehumanizing” clinical practice?

In light of these recent changes in health care, one may anticipate the impact of data science and its medical applications on clinical practice and doctor–patient relationships. At first glance, it is tempting to see such disruptive new technologies as a factor for an evermore dehumanized medicine, where doctors–patient relationships would come down to sensors and computer screens. Based on remote monitoring signals providing detailed clinical information, machine learning algorithms could, for example, display risks of various patient outcomes for each hypothetical therapeutic strategy. Ultimately, physicians may no longer require direct interaction with their patients to accurately assess their clinical and even psychological status. Conversely, one can argue that the progressive automation of various tasks of clinical practice could free up more time for physicians to invest in an improved doctor–patient relationship. Today, most physicians spend a large amount of time trying to detect potential drug interaction, searching for specific events in the patient’s medical history, and surveying repeated and various lab results. This precious medical time could arguably be better spent with the patient, discussing therapeutic choices, assessing treatment comprehension, or detecting psychological vulnerability. Moreover, continuously used monitoring devices, if correctly deployed, could very well enhance doctor–patient relationships by extending it beyond the walls of the physician’s office. Take the case of type 2 diabetes: today’s protocols recommend for a regular follow-up consult in a predetermined sequence, depending on stage and complications; yet, based on multiple inputs (e.g., glycemia controls, self-reported symptoms, urine test strips, etc.), predictive algorithms could very easily generate and send a warning to the patient’s care-givers; this would allow the physician to instantly decide whether to ask his patient to come for an earlier consult. Such system would continuously adapt rigid pre-established guidelines to a more time-flexible and appropriate consult sequence, thus potentially enhancing patient acceptance while optimizing mobility costs.

Additionally, precision medicine could help physicians recommend the best treatment to a given patient. There is no doubt that physicians will be in competition with machine learning algorithms to accurately diagnose diseases or to recommend optimal treatment to patients. Not because machine learning algorithms will reach human intelligence, but because they can quickly access large amounts of data at much lower cost. However, machine learning algorithms are unlikely to achieve the intelligence needed to build mutual understanding between doctors and patients, necessary to establish a quality trusting relationship, which we know plays a central role in the clinical outcomes.

Moreover, some technical aspects of medicine will probably remain in the hands of physicians and other health professionals. Clinical data collection, through both medical interrogatory and physical examination, is on top of the list. The first one requires the translation from common language to medical symptoms, and even though translation algorithms are developing fast, this specific field might be too much of a “niche” for such technologies to take over just yet. The latter seems even more out of reach of automation. Physical examination includes a wide variety of gestures and sensors (e.g., pressure, temperature, sight, hearing), and it requires a level of dexterity to switch and combine those skills that today’s robots can not match. Some surely are superior in specific tasks, but robotization is not moving forward as quickly as data science, and the rise of affordable machines capable of a full personalized physical examination is still decades away. That being said, physicians have long been pushing for more reproducible, standardized, and comparable measurements in order to establish better protocols, and the predominance of clinical data over the so-called “complementary exams” is fading. After all, human sensors, although complex, might be outperformed by simpler yet more efficient 3D imaging. X-rays reach deeper than the hand, echography sees better than the ear, MRI says more than a conversation. It is a race toward who is a better caregiver, and although physicians are still in the lead thanks to their key role in collecting clinical data, machines are closing in fast.

Additionally, as evidence-based medicine shifted medical professions toward more standardized, specialized, and research-oriented tasks, data science could very well increase this phenomenon with physicians spending less time on the front line and more time developing and improving data-driven medical applications. Since modelization plays a key role in data science, a medical doctor’s expertise in anatomy, physiology, and health-care systems, is going to be crucial in the development of this new generation of tools. Moreover, physicians will be required to understand the potential and limitations of these machine learning algorithms. In the future, multiple solutions for care follow-up of type 2 diabetes or different algorithms recommending a personalized sequence of chemotherapy in lung cancer will be available. Physicians will have to provide patients with their professional expertise and experience of these systems, in order to give recommendations about which algorithms are the most efficient, the same way they are asked today about medications. In this sense, physicians’ role will remain central, filling the gap between their patients and an ever-growing mixture of complex health-care solutions based on machine learning algorithms.

We assume that physicians will respond to the evolution of the profession by investing more in human skills. But it is probable that their technical expertise will remain a key factor to new well-performing data-driven applications. Thus, we would argue that physicians and medical students should not only invest an increasing amount of time in social sciences and humanities to develop communication skills, ethics, and understanding of psychosocial or cultural backgrounds but they should also broaden their field of knowledge toward data science.

## Author Contributions

IL and RV contributed equally to the writing of the original manuscript. D-PN, VPL, and RJ reviewed it and suggested improvements. RV designed the graphical descriptions.

## Conflict of Interest Statement

The authors declare that the research was conducted in the absence of any commercial or financial relationships that could be construed as a potential conflict of interest. The reviewer RK and the handling Editor declared their shared affiliation.

## References

[1] LeCun Y Bengio Y Hinton G. Deep learning. Nature (2015) 521:436–44. 10.1038/nature14539 26017442

[2] Sutskever I Vinyals O Le QV Sequence to sequence learning with neural networks. In: Ghahramani Z Welling M Cortes C Lawrence ND Weinberger KQ, editors. Advances in Neural Information Processing Systems 27. Montreal: Curran Associates, Inc. (2014). p. 3104–12.

[3] Krizhevsky A Sutskever I Hinton GE ImageNet classification with deep convolutional neural networks. In: Pereira F Burges CJC Bottou L Weinberger KQ, editors. Advances in Neural Information Processing Systems 25. Lake Tahoe: Curran Associates, Inc. (2012). p. 1097–105.

[4] Hinton G Deng L Yu D Dahl GE Mohamed AR Jaitly N Deep neural networks for acoustic modeling in speech recognition: the shared views of four research groups. IEEE Signal Process Mag (2012) 29:82–97. 10.1109/MSP.2012.2205597

[5] Marchetti MA Codella NCF Dusza SW Gutman DA Helba B Kalloo A Results of the 2016 international skin imaging collaboration international symposium on biomedical imaging challenge: comparison of the accuracy of computer algorithms to dermatologists for the diagnosis of melanoma from dermoscopic images. J Am Acad Dermatol (2017) 78(2):270–7.e1. 10.1016/j.jaad.2017.08.016 28969863 PMC5768444

[6] Gulshan V Peng L Coram M Stumpe MC Wu D Narayanaswamy A Development and validation of a deep learning algorithm for detection of diabetic retinopathy in retinal fundus photographs. JAMA (2016) 316:2402–10. 10.1001/jama.2016.17216 27898976

[7] Rajpurkar P Hannun AY Haghpanahi M Bourn C Ng AY Cardiologist-Level Arrhythmia Detection with Convolutional Neural Networks. *arXiv [cs.CV]* (2017). Available from: http://arxiv.org/abs/1707.01836 (Accessed: March 21, 2018).

[8] Esteva A Kuprel B Novoa RA Ko J Swetter SM Blau HM Dermatologist-level classification of skin cancer with deep neural networks. Nature (2017) 542:115–8. 10.1038/nature21056 28117445 PMC8382232

[9] Feinleib D The Big Data Landscape. Big Data Bootcamp. Berkeley, CA: Apress (2014). p. 15–34.

[10] Mittelstadt BD Floridi L. The ethics of big data: current and foreseeable issues in biomedical contexts. Sci Eng Ethics (2016) 22:303–41. 10.1007/s11948-015-9652-2 26002496

[11] Zalewski A Long W Johnson AEW Mark RG Lehman L-WH. Estimating patient’s health state using latent structure inferred from clinical time series and text. IEEE EMBS Int Conf Biomed Health Inform (2017) 2017:449–52. 10.1109/BHI.2017.7897302 28630952 PMC5473944

[12] Barak-Corren Y Israelit SH Reis BY. Progressive prediction of hospitalisation in the emergency department: uncovering hidden patterns to improve patient flow. Emerg Med J (2017) 34:308–14. 10.1136/emermed-2014-203819 28188202

[13] Finkelstein J Jeong IC. Machine learning approaches to personalize early prediction of asthma exacerbations. Ann N Y Acad Sci (2017) 1387:153–65. 10.1111/nyas.13218 27627195 PMC5266630

[14] Bourke AK Klenk J Schwickert L Aminian K Ihlen EAF Mellone S Fall detection algorithms for real-world falls harvested from lumbar sensors in the elderly population: a machine learning approach. Conf Proc IEEE Eng Med Biol Soc (2016) 2016:3712–5. 10.1109/EMBC.2016.7591534 28269098

[15] Bibault J-E Giraud P Burgun A. Big data and machine learning in radiation oncology: state of the art and future prospects. Cancer Lett (2016) 382:110–7. 10.1016/j.canlet.2016.05.033 27241666

[16] Hsu K-C Wang F-S. Model-based optimization approaches for precision medicine: a case study in presynaptic dopamine overactivity. PLoS One (2017) 12:e0179575. 10.1371/journal.pone.0179575 28614410 PMC5470743

[17] Dente CJ Bradley M Schobel S Gaucher B Buchman T Kirk AD Towards precision medicine: accurate predictive modeling of infectious complications in combat casualties. J Trauma Acute Care Surg (2017) 83:609–16. 10.1097/TA.0000000000001596 28538622

[18] Banerjee A Sanyal D. Dynamics of doctor-patient relationship: a cross-sectional study on concordance, trust, and patient enablement. J Family Community Med (2012) 19:12–9. 10.4103/2230-8229.94006 22518353 PMC3326765

[19] Woolf SH. Practice guidelines: a new reality in medicine. I. Recent developments. Arch Intern Med (1990) 150:1811–8. 10.1001/archinte.150.9.1811 2203320

[20] Farquhar CM Kofa EW Slutsky JR. Clinicians’ attitudes to clinical practice guidelines: a systematic review. Med J Aust (2002) 177:502–6. 12405894 10.5694/j.1326-5377.2002.tb04920.x

[21] Engel GL. The need for a new medical model: a challenge for biomedicine. Science (1977) 196:129–36. 10.1126/science.847460 847460

[22] World Health Organization. Adherence to Long-Term Therapies: Evidence for Action. Geneva: World Health Organization (2003).

[23] Becker MH Patient adherence to prescribed therapies. Med Care (1985) 23:539–55. 10.1097/00005650-198505000-00014 4010350

[24] Connor SR Hospice: Practice, Pitfalls, and Promise. Philadelphia: Taylor & Francis US (1998).

[25] Loscalzo MJ Palliative care: an historical perspective. Hematology Am Soc Hematol Educ Program (2008) 2008:465 10.1182/asheducation-2008.1.465 19074127

[26] McWhinney IR Patient-Centred and Doctor-Centred Models of Clinical Decision-Making. Decision-Making in General Practice. London: Palgrave (1985). p. 31–46.

[27] Declaration on Patient-Centred Healthcare. London, UK: International Alliance of Patients’ Organizations (2006). Available from: https://www.iapo.org.uk/sites/default/files/files/IAPO_declaration_ENG_2016.pdf (Accessed: March 22, 2018).

[28] Alonso Y. The biopsychosocial model in medical research: the evolution of the health concept over the last two decades. Patient Educ Couns (2004) 53:239–44. 10.1016/S0738-3991(03)00146-0 15140464

[29] Horne R Weinman J. Patients’ beliefs about prescribed medicines and their role in adherence to treatment in chronic physical illness. J Psychosom Res (1999) 47:555–67. 10.1016/S0022-3999(99)00057-4 10661603

[30] Waldstein SR Neumann SA Drossman DA Novack DH. Teaching psychosomatic (biopsychosocial) medicine in United States medical schools: survey findings. Psychosom Med (2001) 63:335–43. 10.1097/00006842-200105000-00001 11382261

[31] Charon R Narrative medicine in the international education of physicians. Presse Med (2013) 42:3–5. 10.1016/j.lpm.2012.10.015 23260758 PMC4021016

